# Allergen Immunotherapy: New Insights into an Old Treatment

**DOI:** 10.3390/cells11040679

**Published:** 2022-02-15

**Authors:** Constantinos Pitsios

**Affiliations:** Medical School, University of Cyprus, Nicosia 2109, Cyprus; pitsios.constantinos@ucy.ac.cy

The continuous advancement of biomedical sciences has offered an extreme boost in improving the diagnosis and therapy of allergic diseases. Allergen immunotherapy (AIT) remains the only causative treatment of allergic diseases, aiming to restore immune tolerance. AIT is an example of precision medicine offered by allergologists; after accurate diagnosis of the culprit allergen(s), the appropriate extract or venom is administered. In the more than hundred years of practice in AIT, huge advances have been made in developing novel allergen products for AIT, safety has been increased with the development of depot extracts for subcutaneous immunotherapy (SCIT), with the introduction of sublingual immunotherapy (SLIT) and with new treatment protocols.

In this Special Issue of *Cells*, two up-to-date reviews on AIT are published [1,2]. The humoral and cellular mechanisms involved in AIT are addressed in them. In the article by Pavón-Romero G. et al., an extended report on the use of modified allergens and adjuvants, some of them consisting novel forms of AIT, is offered [1]. Allergoids for SCIT and SLIT have been introduced since 1992 with abundant clinical trials confirming their efficacy. Recombinant allergens have also been designed, with methods being similar to the mRNA vaccines used recently for COVID-19, however, more clinical trials are still needed until they will be commercially available. Demšar Luzar A. et al., have addressed venom immunotherapy (VIT), that after a 3–5 years-long therapy may provide a lifelong tolerance to most patients [2].

Currently, allergen immunotherapy for airborne allergens is practiced with SCIT and SLIT, while VIT is practiced only via SCIT. The intralymphatic route, applying low allergen doses directly into lymph nodes, is a new promising way for AIT addressing respiratory allergies. The latest type of AIT that has been introduced is for food allergy treatment, offering the chance to treat a serious and life-impairing health problem. Various protocols following the oral, the epicutaneous or the—less safe—subcutaneous route, have been studied. For the time being, it appears that oral immunotherapy (OIT) with the “immediately swallowing the allergen” way is the more effective type of food desensitization, followed by the safer SLIT (keeping the allergen under the tongue for a couple of minutes) [3].

An experimental rodent model of OIT, studying the prophylactic and therapeutic effects on allergic conjunctivitis of a hypoallergic tolerogen, is presented in this issue [4]. The idea of this experimental therapy is to develop an OIT method, based on the insertion of the allergen in a staple food, like rice, offering new safe and convenient products for AIT [4]. The outcomes of this OIT should be examined prudently before application to humans, not only due to the short immunization period used in mice, but mostly due to the unusual vector, the route and the experimental allergen used.

Two articles with original data on the experimental production of novel recombinants of the major birch pollen allergen Betv1 have been published by a German team [5,6]. A recombinant fusion protein of flagellin A (serving as a TLR5-ligand) and Betv1, namely rFlaA:Betv1, has been produced, to target immune cells, in order to create adjuvant-mediated pro- and anti-inflammatory responses and suppress the allergen-induced Th2 responses [5]. Using a mouse-model, the stimulation of bone marrow-derived macrophages (BMDMs) by rFlaA:Betv1 and the boosted IL-10 and INF-γ secretion in BMDM and T cell co-cultures, suggest an important role of macrophages as target cells of immunotherapy [5]. The immune-modulatory effects of rFlaA:Betv1 were confirmed in cultures of murine airway LA-4 epithelial cells with myeloid dendritic cells (mDC) [6]. It was shown that, upon stimulation with rFlaA:Betv1, epithelial cells can modulate pro-inflammatory mDC responses by p38 MAPK- and COX2-dependent production of PGE2 [6].

Several health economic studies have been conducted on the cost of SCIT and, despite the heterogeneity of the methodology followed, their common outcome is substantial health care cost savings compared to traditional treatment [7]. An Indonesia-based study evaluating the cost of SCIT in children with allergic rhinitis due to house-dust-mites (HDM) hypersensitivity, is published in this issue [8]. After 18 months of observation, the total health care cost in the SCIT group was estimated slightly lower than in the non-SCIT group [8]. However, an important improvement regarding medication score and “combination of symptom and medication score” was found to be (with statistically significant difference) higher in SCIT group compared to the non-SCIT group. The diagnostic method is a slight limitation of this study; providing HDM monotherapy to 2920 children poses questions on whether other sensitivities were considered.

The identification of one or more allergens that correlates with the suspected triggers and patient exposure into causing the IgE-mediated allergy is the mandatory step before the prescription of AIT. Skin prick tests are the indicated diagnostic method, while the use of serum sIgE is recommended under certain circumstances and the performance of both tests increases the diagnostic sensitivity. A further extended workup is suggested when an AIT candidate presents symptoms or signs suggestive of a disease that is considered a contraindication for AIT [9]. A thorough history and clinical assessment should precede the start of AIT, while complete cell count and tryptase (in cases of moderate-severe sting induced anaphylaxis) are suggested as mandatory [9].

According to the guidelines on allergy diagnosis the performance of intradermal tests (IDT) is currently limited for the assessment of venom hypersensitivity. An article describing the use of IDT for the diagnosis of allergic rhinitis, asthma and/or chronic otitis media with effusion (OME) is published in this issue [10]. It suggests that patients are underdiagnosed with SPT only and that decisions on AIT should include the results of IDT. The efficacy of AIT in patients with low allergen-sensitivity (SPT negative/IDT positive) was found to be similar to the relevant one in patients with high allergen-sensitivity (SPT+IDT positive) [10]. Authors support that the performance of IDT should be assessed in order to detect “allergens clinically relevant to diagnosis of AIT-responsive atopic disease” [10].

The article by Hurst and McDaniel raises some objections. IDT are not recommended by any current national or international consensus as diagnostic tools before AIT. The reason of including groups of patients with OME and chronic eustachian tube dysfunction (ETD) in this study are not clear; these disorders can be related to allergic rhinitis, but do not consist of allergic diseases per se. The authors have included patients with negative SPT and imply that in a subgroup of patients, diagnosis by board-certified allergists was inadequately set, so after being “dissatisfied with their results, these patients sought a second opinion”, resulting IDT positive and started AIT. Unfortunately, the use of the “1-10 Likert scale” cannot guarantee a reliable statistical analysis. Although SPT are not always setting the diagnosis of the culprit allergen with absolute accuracy, better designed studies would be necessary to validate IDT as a diagnostic tool before the decision of AIT.

Advances in immunology including new AIT products and the use of new routes and indications for AIT are challenging. However, the main challenge for physicians should always be to focus on proper training in allergology, including the proper exercise of AIT. AIT is not an “one-size-fits-all” therapy, and Allergologists should offer a precision medicine holistic approach of the patients, treating each one with tailored-made choices.
